# Neighborhood Characteristics Associated with the Availability of Alcohol Outlets in Quebec, Canada

**DOI:** 10.1155/2015/876582

**Published:** 2015-02-24

**Authors:** André Ngamini Ngui, Philippe Apparicio, Mathieu Philibert, Marie-Josée Fleury

**Affiliations:** ^1^Centre de Réadaptation en Dépendance de Montréal, Institut Universitaire, 950 rue de Louvain Est, Montréal, QC, Canada H2M 2E8; ^2^Centre for Research and Intervention on Suicide and Euthanasia (CRISE), Université du Québec à Montréal, 100 Sherbrooke Street West, Montréal, QC, Canada H2X 3P3; ^3^Spatial Analysis and Regional Economics Laboratory, Université du Québec, Institut National de la Recherche Scientifique, Centre Urbanisation Culture Société, 385 rue Sherbrooke Est, Montréal, QC, Canada H2X 1E3; ^4^Bureau d'Information et d'Études en Santé des Populations, Institut National de Santé Publique du Québec (INSPQ), 190 boulevard Crémazie Est, Montréal, QC, Canada H2P 1E2; ^5^Department of Sexology, Université du Québec à Montréal, C.P. 8888, Succursale Centre-ville, Montréal, QC, Canada H3C 3P8; ^6^Department of Psychiatry, McGill University, Douglas Mental Health University Institute Research Centre, QC, Canada

## Abstract

*Objectives*. The objectives of this study were to examine the spatial accessibility to alcohol outlets in Quebec and to assess the association between neighborhood level characteristics and availability of alcohol outlets. *Methods*. The Tobit Model was used to assess the association between neighborhood level characteristics and the availability of alcohol outlets within 500, 1000, 2000, and 3000 metres, respectively. *Results*. Alcohol outlets were found to be most available in the two largest metropolitan areas of the province of Quebec (Montréal and Québec City). Within 1000 metres, alcohol outlets are more available in neighbourhoods with the following characteristics: highest concentration of men, least materially deprived highest concentration of persons aged 20 years or more, and location either in a metropolitan area or in a small town. Finally, the number of bars with video lottery terminals increases with the level of social and material deprivation. *Conclusion*. In Québec, there is no rule governing the location of alcohol outlets. Thus, there is an abundant literature indicating that the regulation of alcohol outlet density could be an effective means of controlling risk attributable to alcohol consumption.

## 1. Introduction

According to the World Health Organization, the global annual consumption of alcoholic beverages per person aged 15 years or older in 2005 was estimated at 6.13 litres of pure alcohol [1]. Recent statistics found that, in 2012, about 17.2% of Canadians aged 12 and over reported heavy drinking [2] whereas another population-based study indicated that the average number of drinks per week among Canadians was 6.4 (7.9 for men and 4.6 for women) [3]. Alcohol consumption is a social and public health issue because it is responsible for many serious social and developmental issues, including violence, assault, self-inflicted injury and homicide [4, 5], child neglect and abuse [6, 7], and absenteeism in the workplace [8, 9]. Alcohol consumption has also been found to be associated with road traffic accidents [10–12], a reduction of social capital [13–15], and poor quality of social networks [16, 17]. Studies consistently show that harmful drinking is a major determinant for neuropsychiatric disorders such as mental health disorders [18], epilepsy [19], and other noncommunicable diseases such as cardiovascular diseases [20–22], cirrhosis of the liver, and various cancers [23, 24]. Alcohol is the world's third largest risk factor for premature mortality (3.2% of all deaths) and is responsible for approximately 4% of disability-adjusted life years in the United States [25]. In Québec, it has been reported that 1.8% of all deaths in 2002 were attributable to alcohol, equivalent to 38,668 life years lost since these deaths occurred mostly among young people. Also, 405,353 episodes of ambulatory care and 48,307 hospitalizations were attributable to alcohol consumption [26].

Greater alcohol outlet density is theoretically and empirically tied to alcohol consumption. One previous study assessing the relationship between alcohol density and the frequent drinking of university and college students, including underage students, found a positive association [27]. Another study reported higher rates of binge drinking among students when there were higher concentrations of alcohol outlets within 1 mile distance of the campus [28]. A meta-analysis conducted by Campbell and colleagues (2009) found a positive association between outlet density and population-level alcohol consumption in five different studies [29]. The association between outlet density and level of alcohol consumption may be explained by the hypothesis that outlet density «*affect excessive alcohol consumption and related harms by changing physical access to alcohol (i.e., either increasing or decreasing proximity to alcohol retailers), thus changing the distance that drinkers need to travel to obtain alcohol or to return home after drinking*» [29].

There is a growing body of research examining the influence of the neighborhood on accessibility of alcohol outlets [4, 30–32]. A greater density of alcohol outlets in deprived neighborhoods has been found in studies from various countries including European and North American [31, 33–35]. In New Zealand, the positive association between neighborhoods' deprivation and alcohol outlet locations has been found in urban areas but not in rural areas [34]. As markers of local population's socioeconomic status (SES), measures of neighborhood-level deprivation generally describe the composition of local populations in terms of income, education, marital status, living arrangements, and so on. Neighborhood level SES is known to be associated with markers of social disorganization, that is to the nature of social relations (e.g., cohesion) and social capital in their ability to regulate individuals' behaviors [36, 37].

However, it is questionable if this association between density of alcohol outlets and deprivation is confounded by other neighborhood characteristics not accounted for (age structure, proportion of immigrants, proportion of people living alone, etc.). Another limitation of these previous studies is that they do not assess the extent to which distance threshold, that is, the distance one is willing to travel to purchase alcohol may influence accessibility to alcohol outlets. Finally, previous studies did not distinguish between types of outlets in the accessibility assessment (e.g., bar-tavern, restaurant, and grocery).

In Canada in general and in Québec in particular, there is a dearth of studies that have directly assessed the association between neighborhood characteristics and the location of alcohol outlets. There is a clear need to explore which factors in the neighborhood attract alcohol outlets in order to control the negative impacts of alcohol outlet locations in the communities. For instance, by controlling the attribution of liquor permits in the neighborhoods in which are located at-risk populations.

The present study had two objectives: (1) to examine the spatial accessibility to alcohol outlets in Québec and (2) to assess the association between neighborhood level characteristics and access to alcohol outlets in general and by type of outlet.

## 2. Methods

### 2.1. Study Location

This research involves the entire province of Québec (Canada) with an area of 1 542 056 km^2^ and about 8.18 million inhabitants at the end of 2013 [38]. In Québec, alcohol can be sold either in outlets administered by the government (*Société des Alcohols du Québec* (SAQ)) or in private outlets licensed by the* Régie des alcools, des courses et des jeux* (RACJ). The RACJ is responsible for administering the* Act respecting liquor permits *as well as the* Act respecting lotteries, publicity contests*,* and amusement machines*. Those who have an alcohol permit can obtain a permit to operate video lottery terminals (VLT). In fact, applicants for a license for video lottery terminals must first possess a license to operate a bar, pub, or tavern [39]. Conversely, all outlets with VTL possess liquor license and sell alcohol, although not all alcohol outlets possess VLT. The register of liquor licenses also includes the type of outlet. The websites of both SAQ and RACJ contain the geographic information (i.e., street address, municipality, and postal code) on the location of each alcohol outlet.

### 2.2. Data Collection Procedure

In November 2013, we accessed the constantly updated websites of RACJ and SAQ from which listings of alcohol outlets were extracted. In total, the database included 19,620 business addresses, of which 270 (1.4%) were removed because they could not be geocoded. It is worth noting that multiple outlets could be located at the same address, for example, a restaurant and a bar. Finally, the 19,350 geocoded addresses (98.6%) contain 24,190 outlets classified in six categories: (1) SAQ outlets (*n* = 400), (2) bars/breweries/taverns (*n* = 5,726), (3) bars with video lottery terminals (*n* = 1,991), (4) restaurants (*n* = 7,782); (5) off-premise (off-premise outlets are not administered by SAQ) (*n* = 7,605), and (6) others (retailers, wholesalers, cider sellers, etc.) (*n* = 686). Alcohol outlets were geocoded using the (*x*, *y*) coordinates of residential or commercial property parcel points.

### 2.3. Area-Level Characteristics

Neighborhood characteristics were obtained from the 2006 Canadian census at the level of the dissemination area (DA) which is the smallest census unit for which socioeconomic and demographic data are disseminated (mean = 583 inhabitants; SD = 368) [40]. Neighborhoods were described in terms of gender composition (percentage of males), ethnic composition (percentage of recent immigrants; i.e., immigrants who arrived in Canada in the last five years), residential instability (percentage of individuals who moved during the last year), age composition (percentage of individuals by age group), area-level socioeconomic status, and an urban-rural denomination.

Area-level socioeconomic status was operationalized using a deprivation index obtained from a principal component analysis consisting of six census variables, which lead to two factors: material and social deprivation [41, 42]. Material deprivation is mainly associated with average income, the unemployment rate and the proportion of persons without a high school diploma. Social deprivation is mostly correlated with the proportion of single-parent families, the proportion of people living alone, and the proportion of persons who are separated, divorced, or widowed. For the purpose of this study, DAs were classified into population weighted quintiles on each type of deprivation, the first quintile representing the least deprived group.

DAs were also categorized based on an urban-rural denomination produced by Statistics Canada. A modification of the Statistical Area Classification (SAC) [43] led to a three-level categorization: metropolitan areas (municipalities with an urban core population ≥100,000 inhabitants), agglomerations (municipalities with a population ranging from 10,000 to 100,000 inhabitants), and rural areas (municipalities with less than 10,000 inhabitants) [44].

### 2.4. Data Analysis

Geographic accessibility was operationalized by the number of alcohol outlets located within a given distance. We used different threshold measures (500, 1000, 2000, and 3000 metres) and this was done for each type of alcohol outlet considered separately as well as for all outlets together. All accessibility measures were first computed at the street block level and then aggregated at the DA level (street blocks are nested within Das) using the population-weighted average number of outlets of each census block. Compared to accessibility measured at the DA level directly, this helped to avoid aggregation errors [45]. For example, the number of alcohol outlets within one kilometre for DA_*i*_ is obtained as follows:

(1) $$\begin{array}{c} A_{\text{D}{\text{A}}_{i}}=\frac{\sum_{b\in i}W_{b}\sum_{j\in S}S_{j}}{\sum_{b\in i}W_{b}}, \end{array}$$

where *W*_*b*_ is the total population of census block *b* within DA_*i*_ and *S*_*j*_ represents the number of alcohol outlets within 1000 metres of block *b*. All distances were computed in ArcGIS using the network analyst extension and the road network was provided by* Adresses* Québec [46].

Next, the Tobit Model was used to assess the association between neighborhood level characteristics and accessibility measures to alcohol outlets in general and by type of outlet. The Poisson regression model is often used with count data. Here, however, such a model was not suitable as the number of outlets had decimal parts due to the aggregation from block-level frequencies to DA-level frequencies of outlets. Considering the large number of DAs with no alcohol outlet, it was difficult to use a standard ordinary least squares (OLS) regression to examine neighborhood characteristics associated with the number of outlets because a simple OLS is likely to yield inconsistent and biased results in such a situation [47, 48]. Because the OLS estimators are always biased downward [49], it has been suggested that applying it to the positive observations is not a satisfactory solution since it does not solve the problem of inconsistency. It would rather introduce an element of selection bias [50]. Therefore, a left censoring mechanism with a minimum value of 0 alcohol outlet was introduced. To account for this censoring, the Tobit regression model (with the SAS QLIM procedure) is used with the number of alcohol outlets available as the response variable. The analyses were weighted by the 2006 total population of each DA. Before proceeding with multivariate analyses, all variables were screened for statistical assumption violations, as well as for missing values and outliers. The correlation coefficient was tested between all variables to avoid multicollinearity in the model [51].

## 3. Results

### 3.1. Availability of Alcohol Outlets in the Immediate Surroundings

Table 1 presents the number of alcohol outlets available within 500, 1000, 2000, and 3000 metres by DA. The average number of alcohol outlets varied from 4.20 within 500 metres to 15.94, 55.68, and 114.60 within one, two, and three kilometres, respectively. Moreover, within 500 metres, 25% of the DAs have 3.75 or more alcohol outlets (see the third quartile). The results also indicate that there are more restaurants and off-premise outlets with alcohol permits whatever the threshold distance. Within 500 metres, there is a mean of 1.52 restaurants, 1.46 off-premise outlets and .71 bar/brewery or tavern available at 500 metres, and these numbers increase significantly to 20.71, 17.89, and 10.11 at 2 kilometres to reach 43.09, 36.31, and 21.07, respectively, at 3 kilometres.

**Figure 1:**
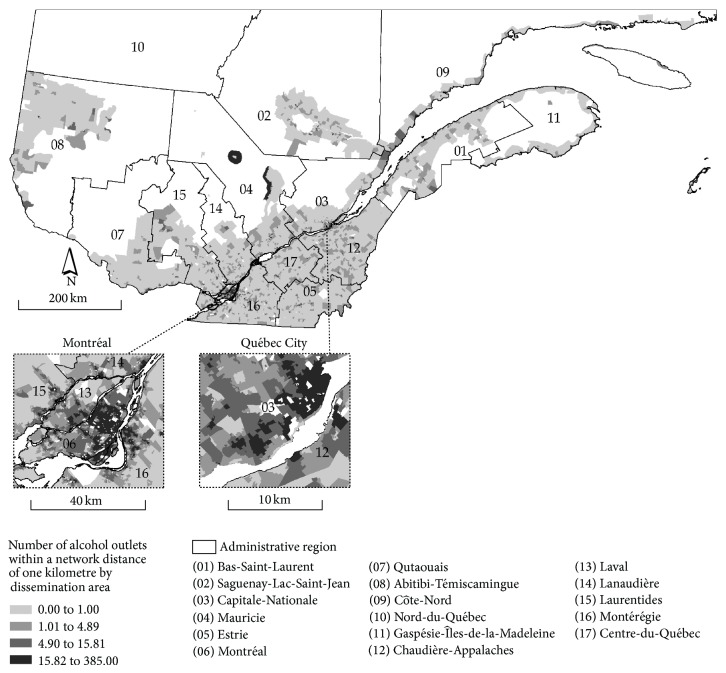
Spatial locations of alcohol outlets in Québec.

For reasons of brevity, we only present the spatial distribution of one accessibility measure, that is, the number of alcohol outlets located within one kilometre of a given DA (Figure 1). It is not surprising that highest values are observed in the two largest metropolitan areas of the province of Québec (Montreal and Québec City). It should be noted that similar spatial patterns are observed for each type of alcohol outlet and each distance threshold (not shown).

### 3.2. Neighborhood Factors Associated with the Availability of All Types of Alcohol Outlets

Table 2 presents the association between neighborhood characteristics and the availability of alcohol outlets in the DAs by distance and type of outlet. All factors were associated with location of outlets, which is due to the high statistical power resulting from the almost complete coverage of our datasets combined with the robustness of the Tobit model [52]. At the threshold of 1 km, a one-percent increase in the number of persons aged between 20 and 34 years is associated with an increase of 3.39 (*P* < .0001) alcohol outlets in general whereas the number of alcohol outlets increases to 1.95 (*P* < .0001) for each one percent increase in the number of persons aged 35–44 in the DA. Within 1 km, the first quintile of material deprivation (least deprived) has 5.89 (*P* < .0001) more alcohol outlets in comparison with the fifth quintile (most deprived). In contrast, social deprivation tends to be inversely associated with the number of alcohol outlets. The findings of Table 2 confirm the highest concentration of alcohol outlets in metropolitan and small towns compared to rural areas. At 1 km for instance, there are 5.07 and 4.44 more alcohol outlets in general respectively in metropolitan areas and small towns than in rural areas. Moreover, the percentage of immigrants in the total population of the DA is also associated positively with all alcohol outlets (*β* = .43, *P* < .0001). Globally, the associations observed at 2 and 3 kilometres are similar but stronger.

### 3.3. Neighborhood Factors Associated with Availability of Specific Types of Alcohol Outlets

As shown in Table 2, at 3 kilometres, metropolitan areas (*β* = 1.66, *P* < .0001) and less materially deprived neighborhoods (*β* = 1.23, *P* < .0001) are more correlated with the SAQ. However, a one-percent increase in the number of immigrants is associated with an increase of 1.54 (*P* < .0001) restaurants and 1.08 (*P* < .0001) off-premise outlets. Compared to rural areas, there are more bars/breweries in small towns and in metropolitan areas at 2 kilometres with 6.24 (*P* < .0001) and 4.72 (*P* < .0001) outlets, respectively. At the same threshold, there are also more bars with video lottery terminal in metropolitan and small towns than in rural remotes with 4.56 (*P* < .0001) and 3.57 (*P* < .0001) outlets, respectively.

Accessibility to alcohol outlets was associated linearly with the level of neighborhood deprivation. At 2 kilometres for instance, accessibility to alcohol outlets was highest in least materially deprived neighborhoods (*β* = 30.87) and lowest in the highest material deprived neighborhood (*β* = −3.92). Inversely, high socially deprived neighborhoods tended to be more accessible to alcohol outlets (*β* = −6.52) than least socially deprived neighborhoods (*β* = −21.61).

At 2 kilometres, every one percentage increase in the number of immigrants in a neighborhood increases the number of off-premise outlets by .48 (*P* < .0001). The number of off-premise outlets also increases for every one percent increase in each age group except for 15–19 years: 2.55 (*P* < .0001) for 20–34 years; 1.88 (*P* < .0001) for 35–44 years; .87 (*P* < .0001) for 45–64 years; and 1.24 (*P* < .0001) for 65 years and over.

The association between off-premise outlets and materially deprived DAs seems more complex. At 500 metres, the most affluent DAs tend to be less attractive and the number of off-premise outlets tends to increase gradually with the level of material deprivation (from *β* = −1.36 to *β* = −.58). As the distance threshold increases, the relation is reverse so that, at 3 Kilometres, more off-premise outlets in the most affluent DAs compare with more deprived (from *β* = .48 to *β* = −4.44). This apparent paradoxical situation may be explained by the fact that (1) as the distance increases, the suburbs are included in the analyses and (2) most of the time, off-premise outlets are included in supermarkets which tend to locate mostly in central cities to be closer to the high concentration of the population.

The association between materially deprived DAs and bars with VLT tends to follow the same pattern as the location of off-premise outlets. Generally, bars with VLT tend to be concentrated in the downtown area and in pericentral districts with the highest population density.

Finally, whatever the distance threshold, it is important to note that the number of bars with video lottery terminals increases with the level of social deprivation. In other words, there are more bars with video lottery terminals in the most socially deprived DAs.

## 4. Discussion

Alcohol outlets provide jobs and have a nonneglected contribution to the economy. Nevertheless, problematic behaviours and important social problems are associated presence of multiple outlets in a neighborhood. To our knowledge, few authors have examined the spatial accessibility of alcohol outlets either in Canada, or in any other developed country. There is a need to document the spatial location of alcohol outlets and the accessibility of neighborhoods to these outlets because it is well established that the availability of alcohol outlets has social consequences such as crime [5], heavy drinking behavior [53, 54], alcohol-related hospital admissions [55], and health problems [56, 57]. Alcohol availability has also been shown as a predictor of youth drinking and driving [58]. The findings of the present study show that accessibility to alcohol outlets in Québec varies by neighborhood characteristics and accessibility is high in metropolitan and small towns. This seems normal given that these areas have the highest population concentration. In fact, it is obvious that owners of alcohol outlets prefer to locate their activity where demand is already high in order to capitalize on demand. Previous studies revealed that the highest clustering of outlets may promote lower prices through discounting and promotion of alcohol products which can entice buyers to consume liquor because of the attractively lower prices [59, 60].

One of the objectives of the study was to examine the relationship between neighborhood characteristics and the location of outlets. Some previous authors have reported a higher concentration of alcohol outlets in poor neighborhoods [4, 34, 61]. Their studies focused only on urban areas whereas the present study includes both rural and urban areas. This may explain in part why we found that whatever the threshold considered, outlets are more located in the most affluent materially deprived neighborhoods.

According to Statistics Canada [62], the prevalence of heavy drinking by age group and sex among Canadians aged 12 years and above in 2012 was 68.87% for men, 7.19% for persons aged 19 years and below, 43.3% among those aged 20–34, 17.16% among 34–44 year olds, 27.39% for 45–64 year olds, and 4.95% for those aged 65 years and above. The spatial location of outlets in Québec tends to follow this pattern. We hypothesized that reducing the density of alcohol outlets in these areas may reduce problematic drinking for these subgroups.

The association between materially deprived neighborhood and concentration of alcohol outlets is an important key finding of this study. Less materially deprived neighborhoods are characterised by an increased presence of restaurants and bar/brewery/tavern while material deprivation is positively associated with the density of off-premise outlets and of bars with lottery terminals. Authors called these two latter types of alcohol outlets “bad bars” because their activities generated neighborhood disturbances such as music and loitering in late hours of the night [63, 64]. Literature indicates that these bad bars tend to be concentrated in crowded, highly dense and disorganized neighborhoods [63–65].

In one previous study on the accessibility of video lottery terminals (VLT) in Montreal, the authors found that VLTs were concentrated in the downtown area and in pericentral districts with the highest population density. They also found a positive correlation between accessibility to VLT and vulnerable neighborhoods [39]. The present study confirms these findings since the number of outlets with VLTs is greater in metropolitan areas followed by small towns. Also, social and material deprivations are positively correlated with the number of outlets with VLTs.

### 4.1. Limitations and Strengths of the Study

The findings of this study should be considered in light of the following limitations. It is important to note that like many other studies of spatial accessibility, our method only concerns potential spatial accessibility, not revealed access (actual utilization of alcohol outlets). It is possible that residents in areas with high potential spatial accessibility (such as the inner city) may not actually enjoy good access to alcohol outlets because aspatial factors such as socioeconomic factors also play an important role in effective accessibility. Another limitation is the design of the study. Using a cross-sectional design, the present study only assesses the association between neighborhood characteristics and alcohol outlet density at a single point of time, which preclude inference on any causal relation. Further, it does not help in understanding if the neighborhood characteristics “attracted” a greater number of alcohol outlets or if the greater number of alcohol outlets impacted the neighborhoods' characteristics.

This study used neighborhood data from different year (2006) even if outlets data are from 2013. It was not possible to use census data of 2011 because major changes to the Canada census of 2011 greatly affected the validity of the data at fine spatial resolution (geographical scale) such as the one used here. We therefore used data for the previous census (2006) for characterising local areas while only the most recent data on alcohol outlets locations were available (2013; no archival data available). This temporal discrepancy is a limit of this study but we opine that the likelihood of bias is low. We have no data describing changes in neighborhood-level characteristics between 2006 and 2013 for the entire province of Québec. However, a study conducted in the Montreal metropolitan area, which encompasses approximately half of the Québec's population, showed that changes in neighbourhood-level SES were marginal between 1986 and 2006 [66].

We have used novel measures of alcohol accessibility and availability which enrich the prevalent notions of alcohol availability. The ability of GIS to handle large amounts of data over large geographic areas at fine levels of geographic detail makes it ideally suited to measure geographical accessibility to spatial facilities. The use of GIS facilitates the production of geographical accessibility measures that overcome the limitations of traditional statistics based on service-to-population ratio and Euclidian distances.

## 5. Conclusion

The findings of this study reveal that accessibility to alcohol outlets in Québec vary by the characteristics of the neighborhood and also by the type of alcohol outlet. Some specific types of alcohol outlets such as bars/breweries, restaurants and off-premises outlets are more accessible in neighborhoods with highest density of persons aged 20 to 34 years.

The results have shown that the potential spatial accessibility to alcohol outlets varies highly across the Province of Québec. For instance, there are about 16 alcohol outlets within a distance of 1 kilometre (SD = 10.2). Moreover, the outlet types more accessible at 1 kilometre are as follows: restaurant (mean = 5.8), off-premise (5.27), bar/brewery-tavern (2.87), and bar with video lottery terminal (1.43). Regarding factors associated with the availability of alcohol outlets, our results suggest that accessibility to alcohol outlets increases in metropolitan, least materially and high socially deprived neighborhoods.

Scientific literature indicates that the regulation of alcohol outlet density could be an effective means of controlling the social consequences of alcohol consumption [5, 29, 34, 67]. In Québec, as in other countries, there is no spatial restriction to obtaining a liquor permit. In other words, there is no restriction on the number of outlets per square kilometre or the number of outlets per 1,000 inhabitants within a radius of one kilometre. Given the relationship between outlet density and the multiple consequences in society, it may be possible to introduce restrictions on the number and density of outlets within a certain distance or to reduce the number of alcohol outlets in metropolitan areas and small towns.

Campbell et al. (2009) have proposed four types of alcohol outlet density regulations: these are geographic restrictions consisting in limiting the number of alcohol outlets per specific geographic unit, population-level restrictions by limiting the number of alcohol outlets per population so that the association between outlet density and alcohol consumption will follow the demand curve; commercial restrictions which aim is toestablish a cap on the percentage of retail alcohol outlets per total retail businesses in a geographic area and; the time/space restrictions which consists of limiting the location and operating hours of alcohol outlets [29]. Bans against alcoholic beverage which consists of reducing the density of alcohol outlets to zero has also been experimented in northern Canada and the southwestern U.S. [68–70].

## Figures and Tables

**Table 1:** Descriptive statistics of accessibility to alcohol outlets in Québec (*N* = 12,712 DAs).

	All	SAQ	Bar/brewery/tavern	Bar with video lottery terminal	Restaurant	Off-premise	Other types
500 metres							
Mean	4.20	.05	.71	.38	1.52	1.46	.09
SD	10.23	.20	2.64	.96	4.95	2.67	.32
Kurtosis	51.52	21.37	160.95	31.47	58.67	12.17	81.08
Skewness	5.95	4.44	10.26	4.61	6.75	3.16	6.98
Quartile 1	.02	.00	.00	.00	.00	.00	.00
Median	.88	.00	.00	.00	.00	.39	.00
Quartile 3	3.75	.00	.46	.25	1.00	1.61	.00
Maximum	196.00	2.00	75.00	14.00	87.00	27.00	6.71
1 kilometre							
Mean	15.94	.22	2.87	1.43	5.81	5.27	.34
SD	35.00	.48	9.37	2.66	16.56	8.59	.79
Kurtosis	35.95	11.66	82.39	13.99	43.01	8.75	37.13
Skewness	5.35	2.91	8.24	3.27	6.06	2.84	4.77
Quartile 1	1.00	.00	.00	.00	.00	.43	.00
Median	4.89	.00	.61	.22	1.00	2.00	.00
Quartile 3	15.81	.09	2.25	1.76	4.46	5.67	.28
Maximum	385.00	5.00	138.43	24.00	190.00	61.50	10.00
2 kilometres							
Mean	55.68	.83	10.11	4.97	20.71	17.89	1.18
SD	112.11	1.26	28.04	7.90	52.20	28.52	2.01
Kurtosis	30.19	16.06	68.85	9.40	38.06	8.29	19.48
Skewness	4.87	3.29	7.41	2.86	5.62	2.80	3.66
Quartile 1	5.06	.00	.81	.12	1.00	2.00	.00
Median	21.00	.47	3.19	2.00	5.62	7.21	.22
Quartile 3	57.32	1.00	9.04	6.00	17.40	18.44	2.00
Maximum	1248.14	12.00	420.00	60.00	605.11	172.00	20.00
3 kilometres							
Mean	114.60	1.71	21.07	10.06	43.09	36.31	2.36
SD	223.19	2.41	52.70	15.80	101.64	59.01	3.59
Kurtosis	20.50	11.47	41.90	7.63	26.76	7.88	12.56
Skewness	4.16	2.96	5.95	2.72	4.81	2.77	3.06
Quartile 1	10.00	.00	1.98	.93	2.14	3.60	.00
Median	43.34	1.00	7.60	4.22	12.83	14.32	1.00
Quartile 3	112.86	2.00	18.64	11.54	37.11	36.35	3.00
Maximum	1857.00	18.00	540.00	94.00	911.00	339.63	28.00

**Table 2:** Weighted Tobit results for neighborhood factors associated with the availability of alcohol outlets^*^.

	All	SAQ	Bar/brewery/tavern	Bar with video lottery terminal	Restaurant	Off-premise	Other types
	*β* (SE)	*β* (SE)	*β* (SE)	*β* (SE)	*β* (SE)	*β* (SE)	*β* (SE)
500 metres							
Intercept	−67.26 (.11)	−3.79 (.02)	−27.15 (.05)	−8.71 (.02)	−39.49 (.08)	−12.75 (.03)	−4.12 (.02)
% male	.42 (.00)	−.01 (.00)	.19 (.00)	.05 (.00)	.20 (.00)	.04 (.00)	.01 (.00)
% 15–19 years	.17 (.00)	.02 (.00)	.08 (.00)	.01 (.00)	.02 (.00)	.02 (.00)	−.01 (.00)
% 20–34 years	1.06 (.00)	.05 (.00)	.33 (.00)	.12 (.00)	.59 (.00)	.27 (.00)	.05 (.00)
% 35–44 years	.61 (.00)	.03 (.00)	.18 (.00)	.07 (.00)	.33 (.00)	.16 (.00)	.03 (.00)
% 45–64 years	.33 (.00)	.02 (.00)	.12 (.00)	.04 (.00)	.22 (.00)	.07 (.00)	.02 (.00)
% 65 years+	.64 (.00)	.04 (.00)	.25 (.00)	.09 (.00)	.38 (.00)	.14 (.00)	.04 (.00)
% mobility	.00 (.00)	.00 (.00)	.02 (.00)	.00 (.00)	.01 (.00)	−.02 (.00)	.00 (.00)
% immigrants	.12 (.00)	.00 (.00)	.02 (.00)	.02 (.00)	.08 (.00)	.04 (.00)	.00 (.00)
Material deprivation							
1st quintile	−.75 (.01)	.21 (.00)	−.07 (.01)	−1.19 (.00)	.69 (.01)	−1.36 (.00)	−.17 (.00)
2nd quintile	−2.03 (.01)	.06 (.00)	−.72 (.01)	−.84 (.00)	−.45 (.01)	−1.2 (.00)	−.19 (.00)
3rd quintile	−1.8 (.01)	.02 (.00)	−.47 (.01)	−.52 (.00)	−.47 (.01)	−.88 (.00)	−.15 (.00)
4th quintile	−1.32 (.01)	.00 (.00)	−.36 (.01)	−.33 (.00)	−.38 (.01)	−.58 (.00)	.00 (.00)
5th quintile	Reference	Reference	Reference	Reference	Reference	Reference	Reference
Social deprivation							
1st quintile	−2.95 (.02)	−.23 (.00)	−1.40 (.01)	−1.09 (.00)	−1.93 (.01)	−1.38 (.00)	−.58 (.00)
2nd quintile	−2.62 (.01)	−.26 (.00)	−1.18 (.01)	−.90 (.00)	−1.48 (.01)	−1.18 (.00)	−.49 (.00)
3rd quintile	−1.4 (.01)	−.04 (.00)	−.52 (.01)	−.47 (.00)	−.6 (.01)	−.75 (.00)	−.31 (.00)
4th quintile	.01 (.01)	.02 (.00)	.06 (.01)	−.08 (.00)	.27 (.01)	−.16 (.00)	−.03 (.00)
5th quintile	Reference	Reference	Reference	Reference	Reference	Reference	Reference
Location of the DA							
Metropolitan area	1.09 (.01)	−.03 (.00)	−.71 (.01)	.41 (.00)	−.14 (.01)	.84 (.00)	.01 (.00)
Small town	.98 (.01)	−.16 (.00)	.26 (.01)	.34 (.00)	−.18 (.01)	.32 (.00)	.21 (.00)
Rural	Reference	Reference	Reference	Reference	Reference	Reference	Reference

1 KILOMETRE							
Intercept	−214.62 (.32)	−3.42 (.01)	−75.14 (.12)	−17.56 (.03)	−110.19 (.18)	−36.48 (.07)	−6.31 (.02)
% Male	1.42 (.00)	−.01 (.00)	.58 (.00)	.09 (.00)	.60 (.00)	.10 (.00)	.01 (.00)
% 15–19 years	.52 (.01)	.02 (.00)	.32 (.00)	.05 (.00)	.22 (.00)	.03 (.00)	.02 (.00)
% 20–34 years	3.39 (.00)	.07 (.00)	.97 (.00)	.27 (.00)	1.67 (.00)	.81 (.00)	.10 (.00)
% 35–44 years	1.95 (.01)	.03 (.00)	.40 (.00)	.18 (.00)	.96 (.00)	.55 (.00)	.05 (.00)
% 45–64 years	1.09 (.00)	.03 (.00)	.34 (.00)	.09 (.00)	.61 (.00)	.25 (.00)	.04 (.00)
% 65 years+	1.87 (.00)	.04 (.00)	.64 (.00)	.19 (.00)	.97 (.00)	.40 (.00)	.06 (.00)
% mobility	−.08 (.00)	.00 (.00)	.04 (.00)	−.02 (.00)	.00 (.00)	−.07 (.00)	.00 (.00)
% immigrants	.43 (.00)	.01 (.00)	.08 (.00)	.04 (.00)	.23 (.00)	.13 (.00)	.02 (.00)
Material deprivation							
1st Quintile	5.89 (.04)	.31 (.00)	2.43 (.02)	−1.42 (.00)	5.65 (.02)	−1.86 (.01)	−.16 (.00)
2nd Quintile	−.60 (.04)	.10 (.00)	−.12 (.01)	−1.14 (.00)	1.79 (.02)	−1.92 (.01)	−.20 (.00)
3rd Quintile	−1.53 (.04)	.05 (.00)	.08 (.01)	−.61 (.00)	.79 (.02)	−1.43 (.01)	−.13 (.00)
4th Quintile	−1.86 (.04)	.06 (.00)	−.04 (.01)	−.38 (.00)	.39 (.02)	−1.00 (.01)	.00 (.00)
5th Quintile	Reference	Reference	Reference	Reference	Reference	Reference	Reference
Social deprivation							
1st Quintile	−6.47 (.05)	−.31 (.00)	−2.47 (.02)	−1.83 (.01)	−3.11 (.03)	−3.21 (.01)	−.72 (.00)
2nd Quintile	−7.22 (.04)	−.35 (.00)	−2.11 (.02)	−1.49 (.00)	−3.08 (.02)	−3.2 (.01)	−.59 (.00)
3rd Quintile	−5.11 (.04)	−.22 (.00)	−1.15 (.02)	−.89 (.00)	−1.74 (.02)	−2.26 (.01)	−.39 (.00)
4th Quintile	−.58 (.04)	.02 (.00)	.22 (.01)	−.14 (.00)	.39 (.02)	−.54 (.01)	−.02 (.00)
5th Quintile	Reference	Reference	Reference	Reference	Reference	Reference	Reference
Location of the DA							
Metropolitan area	5.07 (.04)	.16 (.00)	.16 (.01)	1.49 (.00)	1.51 (.02)	2.99 (.01)	.49 (.00)
Small town	4.44 (.04)	−.06 (.00)	1.85 (.02)	1.19 (.00)	1.27 (.02)	1.21 (.01)	.67 (.00)
Rural	Reference	Reference	Reference	Reference	Reference	Reference	Reference

2 KILOMETRE							
Intercept	−684.36 (.95)	−6.92 (.02)	−197.52 (.29)	−38.76 (.08)	−329.61 (.49)	−123.08 (.22)	−12.53 (.03)
% Male	4.86 (.01)	.00 (.00)	1.56 (.00)	.13 (.00)	2.13 (.01)	.42 (.00)	.02 (.00)
% 15–19 years	.74 (.02)	.06 (.00)	.79 (.01)	.09 (.00)	.67 (.01)	−.14 (.01)	.06 (.00)
% 20–34 years	1.45 (.01)	.13 (.00)	2.49 (.00)	.7 (.00)	4.86 (.01)	2.55 (.00)	.2 (.00)
% 35–44 years	6.43 (.02)	.07 (.00)	1.25 (.01)	.48 (.00)	2.89 (.01)	1.88 (.00)	.12 (.00)
% 45–64 years	3.62 (.01)	.05 (.00)	.97 (.00)	.25 (.00)	1.72 (.00)	.87 (.00)	.09 (.00)
% 65 years+	5.59 (.01)	.08 (.00)	1.52 (.00)	.41 (.00)	2.6 (.00)	1.24 (.00)	.12 (.00)
% mobility	−.47 (.01)	.00 (.00)	.05 (.00)	−.06 (.00)	−.1 (.00)	−.26 (.00)	0 (.00)
% immigrants	1.60 (.00)	.03 (.00)	.27 (.00)	.15 (.00)	.76 (.00)	.48 (.00)	.05 (.00)
Material deprivation							
1st Quintile	28.30 (.12)	.66 (.00)	1.95 (.04)	−.89 (.01)	22.72 (.06)	−1.28 (.03)	.34 (.00)
2nd Quintile	4.40 (.11)	.27 (.00)	3.51 (.03)	−.91 (.01)	8.44 (.06)	−3.23 (.03)	−.06 (.00)
3rd Quintile	1.57 (.11)	.23 (.00)	2.38 (.03)	−.27 (.01)	5.42 (.06)	−1.98 (.03)	.02 (.00)
4th Quintile	−3.76 (.11)	.12 (.00)	.67 (.03)	−.25 (.01)	1.06 (.06)	−2.04 (.02)	.05 (.00)
5th Quintile	Reference	Reference	Reference	Reference	Reference	Reference	Reference
Social deprivation							
1st Quintile	−2.66 (.14)	−.44 (.00)	−5.14 (.04)	−3.26 (.01)	−6.78 (.07)	−9.29 (.03)	−.9 (.00)
2nd Quintile	−26.46 (.13)	−.57 (.00)	−6.08 (.04)	−3.25 (.01)	−9.86 (.07)	−1.43 (.03)	−.91 (.00)
3rd Quintile	−2.88 (.12)	−.43 (.00)	−4.54 (.04)	−2.21 (.01)	−7.07 (.06)	−8.26 (.03)	−.61 (.00)
4th Quintile	−5.88 (.11)	−.05 (.00)	−.74 (.03)	−.35 (.01)	−1.05 (.06)	−2.86 (.03)	−.1 (.00)
5th Quintile	Reference	Reference	Reference	Reference	Reference	Reference	Reference
Location of the DA							
Metropolitan area	17.55 (.11)	.78 (.00)	4.72 (.03)	4.56 (.01)	7.16 (.06)	9.11 (.03)	1.48 (.00)
Small town	12.38 (.13)	.32 (.00)	6.24 (.04)	3.57 (.01)	6 (.07)	3.06 (.03)	1.72 (.00)
Rural	Reference	Reference	Reference	Reference	Reference	Reference	Reference

3 KILOMETRE							
Intercept	−1335.6 (1.81)	−12.14 (.02)	−364.31 (.50)	−68.76 (.14)	−645.96 (.90)	−244.6 (.45)	−19.10 (.04)
% Male	90.57 (.03)	.02 (.00)	2.90 (.01)	.16 (.00)	4.48 (.01)	.98 (.01)	.04 (.00)
% 15–19 years	.43 (.04)	.07 (.00)	.97 (.01)	.06 (.00)	.96 (.02)	−.61 (.01)	.07 (.00)
% 20–34 years	2.77 (.02)	.22 (.00)	4.81 (.01)	1.34 (.00)	9.49 (.01)	5.00 (.00)	.32 (.00)
% 35–44 years	12.88 (.03)	.13 (.00)	2.59 (.01)	.98 (.00)	5.76 (.02)	3.65 (.01)	.18 (.00)
% 45–64 years	6.97 (.02)	.08 (.00)	1.74 (.00)	.47 (.00)	3.21 (.01)	1.67 (.00)	.12 (.00)
% 65 years+	1.79 (.02)	.13 (.00)	2.73 (.00)	.72 (.00)	4.96 (.01)	2.41 (.00)	.18 (.00)
% mobility	−1.23 (.01)	.00 (.00)	.00 (.00)	−.14 (.00)	−.32 (.00)	−.57 (.00)	−.01 (.00)
% immigrants	3.42 (.00)	.06 (.00)	.50 (.00)	.32 (.00)	1.54 (.00)	1.08 (.00)	.09 (.00)
Material deprivation							
1st Quintile	57.14 (.22)	1.23 (.00)	21.33 (.06)	−.14 (.02)	42.49 (.11)	.48 (.06)	.97 (.00)
2nd Quintile	6.21 (.22)	.53 (.00)	6.71 (.06)	−1.02 (.02)	14.60 (.11)	−5.35 (.05)	.20 (.00)
3rd Quintile	1.18 (.21)	.39 (.00)	4.23 (.06)	−.22 (.02)	8.90 (.10)	−3.63 (.05)	.22 (.00)
4th Quintile	−10.03 (.20)	.16 (.00)	.75 (.06)	−.63 (.02)	.79 (.10)	−4.44 (.05)	.05 (.00)
5th Quintile	Reference	Reference	Reference	Reference	Reference	Reference	Reference
Social deprivation							
1st Quintile	−35.42 (.26)	−.56 (.00)	−7.17 (.07)	−5.25 (.02)	−1.54 (.13)	−16.79 (.07)	−1.16 (.01)
2nd Quintile	−49.17 (.25)	−.79 (.00)	−9.79 (.07)	−5.48 (.02)	−17.29 (.12)	−19.56 (.06)	−1.3 (.01)
3rd Quintile	−4.43 (.23)	−.59 (.00)	−7.97 (.06)	−4.02 (.02)	−13.51 (.11)	−15.87 (.06)	−.93 (.01)
4th Quintile	−1.46 (.21)	−.05 (.00)	−1.27 (.06)	−.61 (.02)	−2.05 (.10)	−5.55 (.05)	−.13 (.00)
5th Quintile	Reference	Reference	Reference	Reference	Reference	Reference	Reference
Location of the DA							
Metropolitan area	38.52 (.20)	1.66 (.00)	10.58 (.06)	8.53 (.02)	15.24 (.10)	17.67 (.05)	2.74 (.01)
Small town	19.93 (.24)	.90 (.00)	10.37 (.07)	6.15 (.02)	9.26 (.12)	4.98 (.06)	2.82 (.01)
Rural	Reference	Reference	Reference	Reference	Reference	Reference	Reference

^*^All variables are significant at the level of *P* < .0001.
